# Challenges of cardiac sarcoidosis

**DOI:** 10.3389/fmed.2023.999066

**Published:** 2023-03-02

**Authors:** Irina R. Strambu

**Affiliations:** ^1^Pulmonology Department, University of Medicine and Pharmacy “Carol Davila”, Bucharest, Romania; ^2^Institute of Pneumophthysiology “Marius Nasta”, Bucharest, Romania

**Keywords:** sarcoidosis, cardiac sarcoidosis, cardiac MRI, guidelines, corticosteroids

## Abstract

Sarcoidosis is a multisystem granulomatosis of unknown origin, which can involve almost any organ. Most frequently the disease involves the lungs and mediastinal lymph nodes, but it can affect the skin, the eyes, nervous system, the heart, kidneys, joints, muscles, calcium metabolism, and probably any other anecdotical organ involvement. Cardiac sarcoidosis is one of the most challenging involvements, as it can lead to cardiac mortality and morbidity, and also because the diagnosis may be difficult. With no specific symptoms, cardiac sarcoidosis may be difficult to suspect in a patient with no previous extra-cardiac sarcoidosis diagnosis. This manuscript reviews the current knowledge of the diagnosis and decision to treat cardiac sarcoidosis, and illustrates the information with a case presentation of a young adult with no risk factors, no previous diagnosis of sarcoidosis, and with cardiac symptoms impairing his quality of life.

## Introduction

Sarcoidosis is a multisystem granulomatosis of unknown origin, characterized by the formation of epithelioid granulomas in multiple organs in susceptible individuals.

Sarcoidosis involves most frequently the lungs and mediastinal lymph nodes, and about 90% of diagnosed patients have this type of intrathoracic involvement (1).

Sarcoidosis can affect almost any organ in the body, as an isolated involvement, or associated to lung or other organs disease. It can involve lymph nodes.

The heart is one of the organs that can develop the disease and is generally considered as one of the involvements that can be associated to high risk for premature death or irreversible heart conditions. This makes cardiac sarcoidosis one of the biggest concerns for severe disease.

Still, the heart involvement in sarcoidosis may be difficult to recognize in clinical practice, and the prevalence of the disease may be underestimated.

This article reviews the knowledge on the subject and illustrates it with the presentation of a case.

## Prevalence of cardiac sarcoidosis

Sarcoidosis affects about 60 people per 100,000 in the United States, and has variations of prevalence over the world, with Northern Europe and African Americans, having a higher incidence, but with variation of incidence in regions of the same country (2).

Cardiac sarcoidosis was found in pathology studies in up to 30% of patients with sarcoidosis (3), but because in many cases it may be asymptomatic, it is noted typically in about 7% of sarcoidosis patients, with or without involvement of other organs (4).

Cardiac sarcoidosis is associated to mortality rates between 1 to 8%, most of them caused by cardiac involvement (3).

## Heart involvement in sarcoidosis

Sarcoid granulomas may involve any area of the heart (5). Most frequently, the lessions are found in the interventricular septum, especially at the base. Other preferred sites are the left ventricle free wall and right ventricle, the atria being less commonly involved (6).

In patients requiring heart transplantation or who die from sudden death due to cardiac sarcoidosis, extensive scarring can be found, as a consequence of chronic disease (5).

Cardiac structural changes and presence of granulomas may induce the clinical manifestations of the disease: arrhythmias, conductance disturbances, dilated cardiomyopathy, right heart failure and peripheral edema, or left heart failure with pulmonary edema and hypertension, valvular disfunctions, or sudden cardiac death (4).

Clinical presentation includes most commonly: atrioventricular block, ventricular arrhythmias, and heart failure (7). Patients may also present less commonly with valvular abnormalities, bundle branch block, pericardial effusion (8). Pericardial involvement is uncommon, may be an incidental discovery and it may be associated to myocardial lessions in about 25% of cases (9). It is not clear if it is a distinct sarcoidosis serositis (similar to pleural effusion), it is secondary to ventricular failure or it is accompanying the heart involvement (10).

Cardiac sarcoidosis should be suspected in individuals presenting with new and unexplained atrial or ventricular arrhythmias, atrio-ventricular block, or with left ventricular dysfunction. Sometimes the presentation is less specific, with palpitations or presyncope as the only presenting symptoms. Cardiac sarcoidosis can be suspected particularly in patients who have already a diagnosis of sarcoidosis involving other organs. The real problem are the patients presenting with nonspecific symptoms like palpitations and syncope or presyncope, without being previously diagnosed with sarcoidosis. In these individuals, the diagnosis may be significantly delayed (8, 11).

## Diagnosis of cardiac sarcoidosis

Diagnosis of cardiac sarcoidosis can be challenging from several perspectives.

Probably the most challenging issue is the suspicion of CS with initial cardiac manifestations in a patient with no previous diagnosis of sarcoidosis, or no other clinical apparent organ involvement. The sarcoidosis as the cause of heart rhythm disturbances in such a patient may not be suspected by the cardiologist, and diagnosis can be delayed.

Another difficulty in diagnosis is the absence of a golden standard for diagnosis.

Histologic proof of the epithelioid granulomas is part of the definition of sarcoidosis. For the heart involvement, the biopsy of endomyocardial tissue has a high specificity, but sampling errors and variable histology reduce the sensitivity down to 20 to 30% (12). Also, endomyocardial biopsies are usually obtained from the right side of the interventricular septum, while granulomas are typically located in the left ventricle (13).

An initial electrocardiogram may show a variety of abnormalities that are not specific to cardiac sarcoidosis: atrioventricular block type I, II or III, atrial and ventricular arrhythmias, including ventricular premature beats or ventricular tachycardia (8). Holter monitoring, used for detecting arrhythmias or heart block, has a sensitivity of 59 to 67% and a specificity of 58 to 80% (14). Heart block is often an early sign of cardiac involvement and it may be the manifestation with the best chance of responding to corticosteroids (15).

Heart ultrasound is accessible and non-invasive, can be easily performed in patients presenting with cardiac symptoms, but it has little specificity for early or localized cardiac sarcoidosis, and patients with cardiac sarcoidosis may have a normal echocardiogram (16).

When present, abnormal findings include abnormal myocardial wall thickness, possibly due to presence of granulomas, wall motion abnormalities, or diastolic dysfunction. In later stages of the disease, thinning of the myocardial wall, left ventricular dilatation and left ventricular systolic dysfunction may be noticed. These are considered predictors of mortality for cardiac sarcoidosis (16).

Cardiac magnetic resonance study has become the standard of care for the diagnosis of cardiac sarcoidosis. The findings may include regional wall motion abnormalities in a patchy distribution, and regional increased signal intensity on T2 and delayed gadolinium enhancement. The areas most frequently involved for enhancement are the basal interventricular septum. Though, any myocardial segment of the left or right ventricle can be involved: subepicardial, transmural or midmyocardial regions (17, 18).

PET-CT scan can be useful for the diagnosis of sarcoidosis, the whole-body scan being able to locate multiple organ involvements of the disease and allows the choice of the most accessible site for performing a biopsy (19).

PET-CT is useful for the diagnosis of cardiac sarcoidosis, with the use of a cardiac dedicated protocol including both FDG PET (to image the inflammation) and a scan to assess resting myocardial perfusion images with ^82^Rubidium or ^13^N-Ammonia, and using a protocol for preparing the patient with a high fat/low carbohydrate diet before the examination (11). Focal areas of FDG increased uptake may appear, corresponding with areas of resting perfusion defects. The overlap of regions of high FDG uptake and resting perfusion defects can support the diagnosis of cardiac sarcoidosis (11).

The sensitivity of PET-CT for the diagnosis of CS is 89% with a specificity of 78% (20).

Due to the difficulty to use a single golden standard test for the diagnosis of cardiac sarcoidosis, sets of major and minor criteria for the diagnosis were proposed in consensus statements.

The Japanese Ministry of Health and Welfare consensus states that, in the absence of a diagnostic endomyocardial biopsy, a patient with known extra-cardiac sarcoidosis may be diagnosed with cardiac sarcoidosis by fulfilling at least two major criteria or at least one major plus two minor criteria. Major criteria include advanced atrioventricular block, thinning of the basal interventricular septum, positive gallium-67 uptake, or diminished ejection fraction. Minor criteria include abnormal ECG findings, perfusion defects on nuclear imaging, delayed enhancement of the myocardium by cardiac MRI, or endomyocardial biopsy showing interstitial fibrosis or monocyte infiltration (21).

In 2014, the Heart Rhythm Society released a similar consensus statement, adding as criteria the response to corticosteroid or immunosuppressive therapy and the reasonable exclusion of other cause (s) for the cardiac manifestations (22).

The WASOG criteria for the diagnosis of cardiac sarcoidosis, classified according to the organ assessment instrument developed by this society, include (23):

- *Highly probable:* biopsy with granulomatous inflammation of no alternate cause.
- *Probable:*
  o Treatment responsive cardiomyopathy.
  o Atrio-ventricular block.
  o Reduced left ventricle EF in the absence of other clinical risk factors.
  o Spontaneous or inducible sustained ventricular tachyarrhythmia with no other risk factor.
  o Mobitz type II or 3rd degree heart block.
  o Patchy uptake on dedicated cardiac PET.
  o Delayed enhancement on CMR.
  o Positive gallium uptake.
  o Defect on perfusion scintigraphy or SPECT scan.
  o T2 prolongation on CMR.
- *Possible:*
  o Reduced LVEF in the presence of other risk factors (e.g., systemic hypertension, diabetes mellitus).
  o Atrial dysrhythmias.
- *No consensus:*
  o Frequent ectopy (>5% QRS).
  o Bundle branch block.
  o Impaired right ventricle function with a normal pulmonary vascular resistance.
  o Fragmented QRS or pathologic Q waves in ≥2 anatomically contiguous leads.
  o At least one abnormal signal averaged ECG domain.
  o Interstitial fibrosis or monocyte infiltration.

## Prognosis of cardiac sarcoidosis

Cardiac sarcoidosis can be life threatening, as shown by a study in which over a 2.6 years follow-up, 8% of patients were recorded with death, aborted sudden cardiac death, or intra-cardiac device therapy (24). The MRI changes can be considered predictors for the risk of sudden death, with higher risk for patients who have proven late gadolinium enhancement (11). Moreover, it has been shown that extensive late gadolinium enhancement is associated to poor outcome, higher risk of death and severe arrhythmias and lack of improvement of left ventricular function, despite corticosteroid therapy (25).

## When to treat cardiac sarcoidosis?

Cardiac sarcoidosis is potentially an important threat for mortality and long-term cardiac consequences. In the view of the classic “Wells postulate,” to treat sarcoidosis if there is risk or impairment of the quality of life, cardiac sarcoidosis should be considered “risk” and treatment should be started.

Recently, a European Respiratory Task Force committee composed of clinicians, methodologists and patients developed eight specific questions, used to formulate specific evidence-based recommendations. These were developed based on the GRADE methodology (15).

Question 5 refers to cardiac sarcoidosis: “*In patients with clinically relevant cardiac sarcoidosis, should glucocorticoids with or without other immunosuppressives* versus *no immunosuppression be used?”*

The proposed answer is that in patients with evidence of functional cardiac abnormalities, including heart block, dysrhythmias, or cardiomyopathy, it is recommended the corticosteroid therapy (with or without other immunosuppressives) (strong recommendation, very low quality of evidence) (15). The treatment should be recommended only in patients with significant clinical consequences of cardiac involvement, and not to all patients with imaging changes suggestive of cardiac sarcoidosis.

The committee notes that the evidence supporting the use of corticosteroids in cardiac sarcoidosis is indirect. Still, corticosteroid treatment was associated with improvement in left ventricular ejection fraction (26), so the danger associated to cardiac sarcoidosis favors the corticosteroid treatment for clinically relevant cardiac sarcoidosis (15).

The features found to be associated with increased risk of mortality or morbidity that may influence treatment decisions for cardiac sarcoidosis are (15):

- Age greater than 50.
- Left ventricular ejection fraction less than 40%.
- NYHA functional class 3 or 4.
- Increased left ventricular end-diastolic diameter.
- Late gadolinium enhancement on cardiac MRI.
- Ventricular tachycardia.
- Cardiac inflammation identified by FDG-PET scan.
- Interventricular septum thinning.
- Elevated troponin or brain natriuretic peptide.

## Treatment of cardiac sarcoidosis

The current evidence suggests that corticosteroids are effective in reducing the granulomas in the heart, diminishing the dysrhythmias and blocks and improving the ventricular function (26). Still, patients with severe impairment of the ejection fraction (< 30%) may not respond to treatment, probably due to irreversible fibrotic changes in these patients (8).

The current trend to reduce the initial dose of corticosteroids for sarcoidosis treatment is applicable also for cardiac involvement. A retrospective analysis suggested that prednisolone doses higher than 0.5 mg/kg were not more effective than a starting dose of 0.5 mg/kg (27). The duration of treatment was mentioned in several studies between 3 and 168 months (28).

Steroid-sparing drugs most frequently mentioned are methotrexate, azathioprine, mycophenolate mofetil, leflunomide, and cyclophosphamide. These drugs were used also as alternatives in patients refractory to or with major contraindications to corticosteroids (29, 30). The efficiency of these agents, used in combination with low-dose corticosteroids or alone, is similar to corticosteroid treatment, with similar rates of relapse (33–35%), but more dedicated trials to define the optimal combination are needed (31). Recently, TNF-α antagonists (inflizimab, adalimumab) showed promising results in refractory cardiac sarcoidosis patients (32, 33).

## Screening for cardiac sarcoidosis

The current knowledge supports the screening for cardiac sarcoidosis in asymptomatic patients with extra-cardiac sarcoidosis (22). There is still debate regarding the best way to screen for cardiac involvement. Physical examination and medical history specifically asking about palpitations, syncope, and chest pain should be performed in all patients. The American Thoracic guidelines recommend performing routine ECG to screen for the possible cardiac involvement. The routine echocardiography or 24-h continuous ambulatory ECG are not recommended in patients without cardiac symptoms or signs, but could be used on a case-by-case basis (34). Routine echocardiography, mentioned in Heart Rhythm Society, may overlook the cardiac involvement (35). The use of cardiac MRI, rather than cardiac PET, should be recommended for patients with extra-cardiac sarcoidosis that exhibit symptoms of heart involvement, and also to patients with no previous diagnosis of sarcoidosis who present with unexplained Mobitz II or third-degree AV block and sustained monomorphic ventricular tachycardia of unknown cause (8, 34).

## A young adult with palpitations

**Figure 1:**
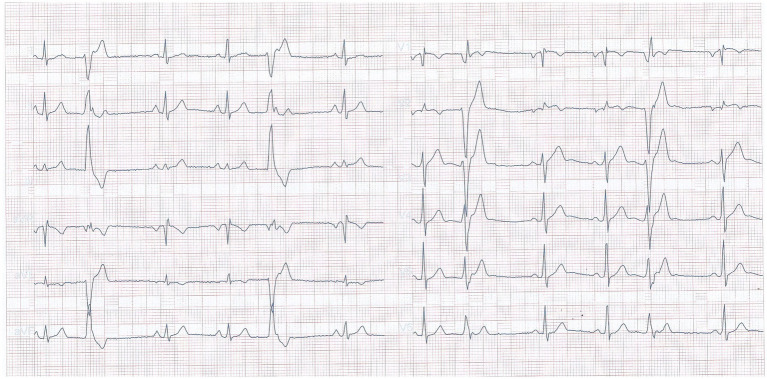
Electrocardiogram recorded during an emergency presentation, showing frequent premature ventricular beats.

**Figure 2:**
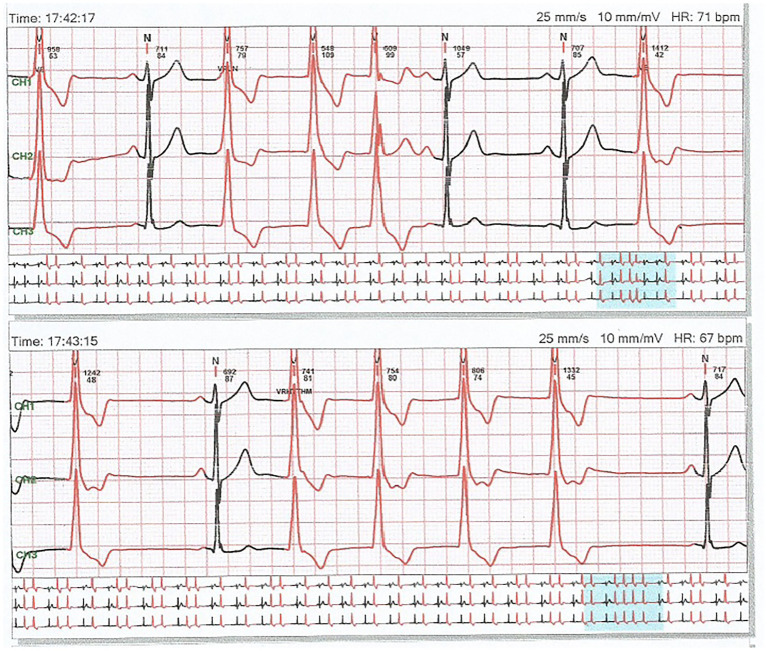
A 24 h recording of electrocardiogram showing frequent premature ventricular beats with unique morphology, mimicking a right bundle branch block.

A 32 years old nonsmoker male patient had a 2 years history of palpitations occurring in episodes, apparently unrelated to any triggering event. The episodes were sometimes accompanied by presyncope, and he presented repeatedly in emergency department during these events. The electrocardiograms were repeatedly showing ventricular arrhythmia, with frequent ventricular premature beats with monophocal pattern, sometimes organized in bigeminism or trigeminism (Figure 1). Heart ultrasound performed on these occasions did not show any change in ventricular function. He consulted a cardiologist specialized in heart rhythm disturbances, and received several antiarhythmic drugs (bisoprolol, sotalol, and propaphenone), which did not prevent the occurrence of new palpitations episodes, accompanied by the same lipotimic state. Continuous 24-h ECG recording showed the presence of premature ventricular beats also outside the clinically manifest episodes (Figure 2). The premature beats had the same morphology, mimicking a right bundle branch block.

A cardiac MRI with gadolinium enhancement was performed, which showed increased thickness of left ventricle during systole and contrast enhancement in the middle of cardiac wall at the base of the heart, and was considered by the radiologist who evaluated it initially as non-obstructive hypertrophic cardiomyopathy.

**Figure 3:**
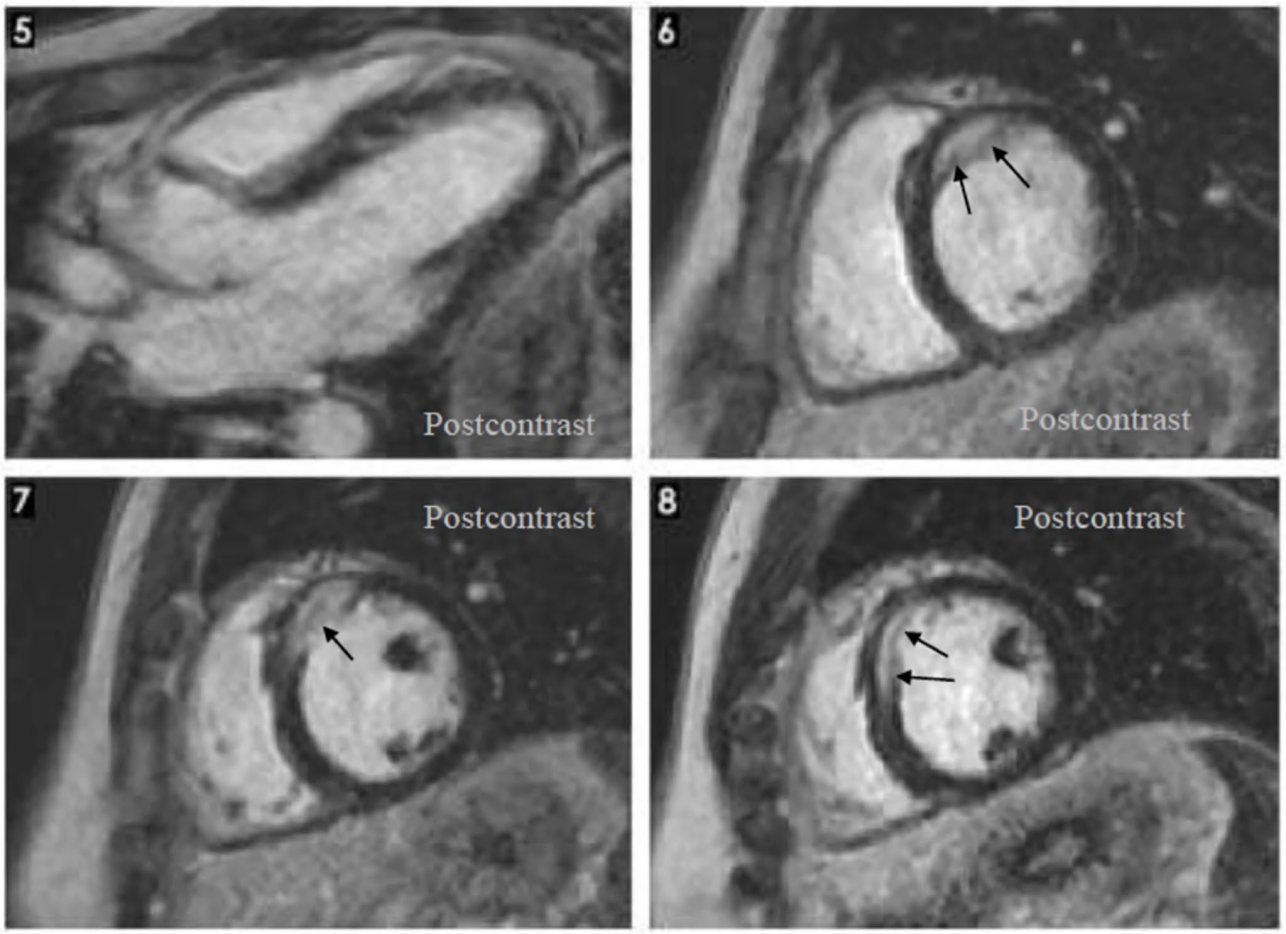
Cardiac magnetic resonance: Hypertrophy localized at the base of the septum, T2 focal edema at the base of the septum, late gadolinium enhancement: homogenous late uptake of gadolinium in the granuloma.

**Figure 4:**
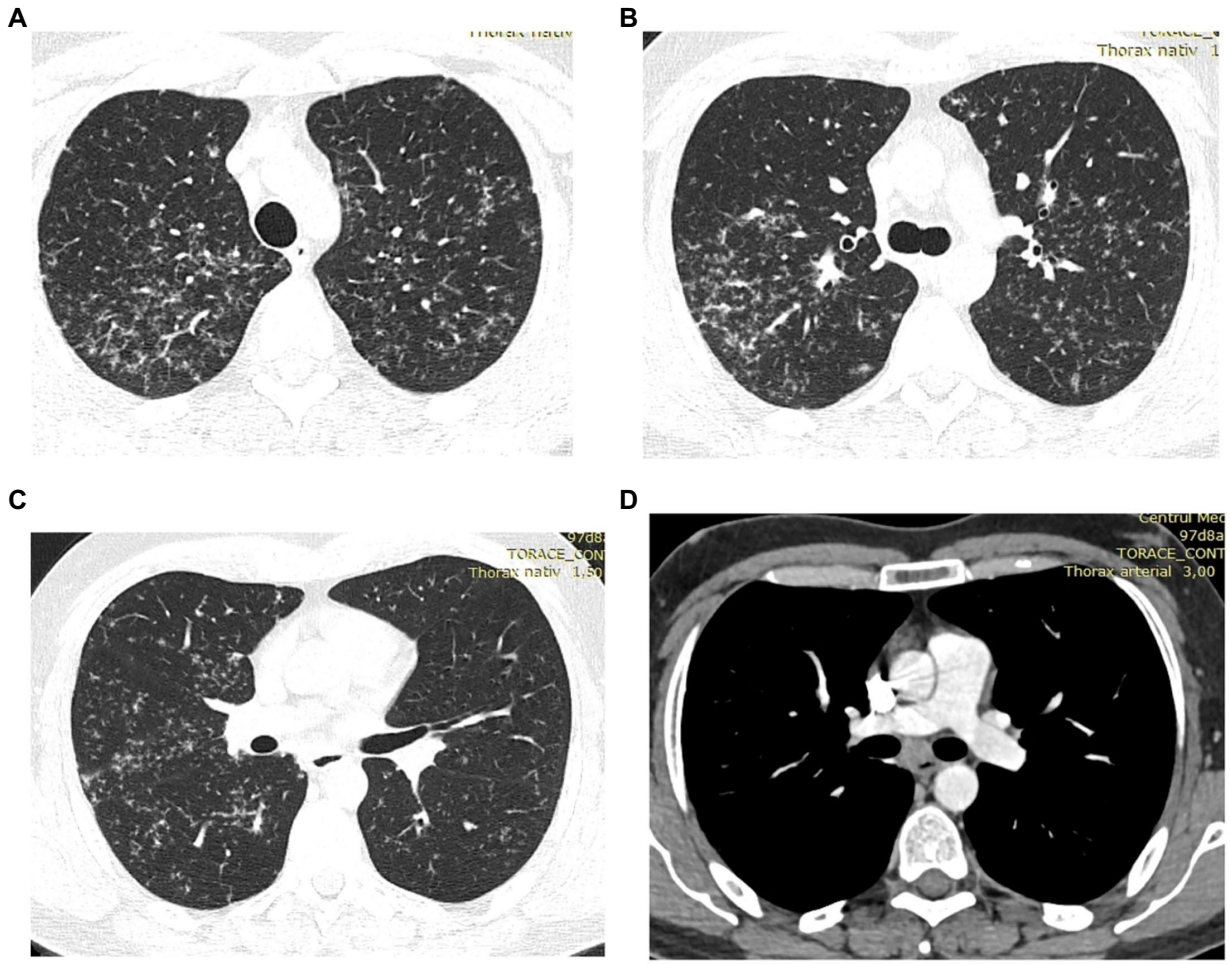
**(A–D)** HRCT of the lungs: multiple bilateral micronodules predominant in the upper lobes, increased infracarinal lymph nodes.

As the patient was not satisfied with the outcome of the investigations and the treatment, he sought a second opinion. On this occasion, the same cardiac MRI images were analyzed by a cardiologist specialized in MRI imaging of the heart. He interpreted the same images differently, describing the presence of focal edema at the base of the septum in T2 and granulomas with homogenous uptake of gadolinium at the base of the interventricular septum (Figure 3). He concluded that the image changes are highly suggestive for cardiac sarcoidosis. He also referred the patient to the pulmonology unit for further evaluation. The CT scan of the thorax showed multiple micronodules distributed mostly in the upper parts of the lungs, with a perilymphatic distribution, as well as moderately enlarged mediastinal lymph nodes (Figure 4). A bronchoscopy was performed, with a transbronchial biopsy of the mediastinal lymph nodes. The histology did not overtly confirm the presence of typical sarcoid granulomas, describing lymphocytic inflammation and some giant cells. Broncho-alveolar lavage proved a lymphocytic alveolitis with 34.4% lymphocytes in the BAL fluid, with normal CD4/CD8 ratio. The serum angiotensin-converting enzyme was highly elevated, three-fold the normal upper limit.

The patient did not complain of any respiratory symptoms, and the lung function tests were normal, including the diffusion capacity. He did not show signs of any other organ involvement: he had normal renal function, normal calcium level in the blood and urine, had no neurological symptoms, no eye ailment, no skin changes, no peripheral lymph node enlargement, and no joint pain.

He was diagnosed with sarcoidosis involving the lungs, mediastinal lymph nodes and heart. The diagnosis was considered as such even in the absence of a clear histologic confirmation. The BAL lymphocytosis was compatible with sarcoidosis (36). The normal CD4/CD8 ratio does not exclude the diagnosis, as it has a limited value (37). In this patient, even in the absence of a typical biopsy, applying the clinical sarcoidosis diagnosis score (SDS clinical) would have brought enough elements in favor of the diagnosis: highly probable: HRCT imaging changes, at least probable: BAL lymphocytic alveolitis, TBNA showing lymphoid aggregates and giant cells, spontaneous ventricular tachyarrhythmia in the absence of other risk factor, delayed enhancement on cardiac MRI (38, 39).

Oral corticosteroid treatment was started at a dose of 32 mg of methylprednisolone per day, tapered to 8 mg per day over 6 months. Low dose treatment was continued up to 36 months. The clinical improvement was noticed during the first month of treatment. The frequency of the palpitation episodes decreased, and the events were no longer accompanied by presyncope. The arrhythmic events disappeared completely during the first year of treatment. Cardiological examinations and chest ultrasound showed no impairment of cardiac function. The corticosteroid treatment was well tolerated, with minimal muscle weakness during the first months of treatment.

This case illustrates the difficulty of diagnosis of cardiac sarcoidosis in a patient that is not previously diagnosed with the disease, based on other organ involvements. Our patient had highly suggestive changes on the HRCT of the lungs, but this was not performed previously as he did not complain of any respiratory symptoms. The frequent visits to the emergency department and the occurrence of severe arrhythmia in a young patient with no risk factors and no other heart problems should have triggered the suspicion of a cardiac sarcoidosis earlier. The patient displayed the late gadolinium enhancement on cardiac MRI, which is considered a risk factor for severe outcome, and had potentially severe ventricular arrhythmia, but fortunately the function of the ventricle was not impaired and responded well to corticosteroid treatment.

Cardiac sarcoidosis can represent a life-threatening condition, the risk being augmented by the lack of suspicion of this disease in patients with no previous diagnosis of sarcoidosis.

## Author contributions

IS contributed to the review of literature, editing the manuscript, medical care of the illustrative case, and choice of the figures. The author confirms being the sole contributor of this work and has approved it for publication.

## Conflict of interest

The author declares that the research was conducted in the absence of any commercial or financial relationships that could be construed as a potential conflict of interest.

## Publisher’s note

All claims expressed in this article are solely those of the authors and do not necessarily represent those of their affiliated organizations, or those of the publisher, the editors and the reviewers. Any product that may be evaluated in this article, or claim that may be made by its manufacturer, is not guaranteed or endorsed by the publisher.
